# Structural and Functional Features of Chars From Different Biomasses as Potential Plant Amendments

**DOI:** 10.3389/fpls.2018.01119

**Published:** 2018-08-17

**Authors:** Marta Marmiroli, Urbana Bonas, Davide Imperiale, Giacomo Lencioni, Francesca Mussi, Nelson Marmiroli, Elena Maestri

**Affiliations:** ^1^Department of Chemistry, Life Sciences and Environmental Sustainability, University of Parma, Parma, Italy; ^2^National Interuniversity Consortium for Environmental Sciences (CINSA), Parma, Italy; ^3^Interdepartmental Center for Energy and Environment (CIDEA), University of Parma, Parma, Italy

**Keywords:** biochar, low-vacuum SEM/EDX, phytotoxicity, principal component analysis, pyrolysis, soil improvers, trace elements

## Abstract

Biochars result from the pyrolysis of biomass waste of plant and animal origin. The interest in these materials stems from their potential for improving soil quality due to increased microporosity, carbon pool, water retention, and their active capacity for metal adsorption from soil and irrigation water. Applications in agriculture have been studied under different conditions, but the overall results are still unclear. Char structure, which varies widely according to the pyrolysis process and the nature of feedstock, is thought to be a major factor in the interaction of chars with soil and their metal ion adsorption/chelation properties. Furthermore, biochar nutrients and their elemental content can modify soil fertility. Therefore, the use of biochars in agricultural settings should be examined carefully by conducting experimental trials. Three key problems encountered in the use of biochar involve (i) optimizing pyrolysis for biomass conversion into energy and biochar, (ii) physicochemically characterizing biochar, and (iii) identifying the best possible conditions for biochar use in soil improvement. To investigate these issues, two types of wood pellets, plus digestate and poultry litter, were separately converted into biochar using different technologies: pyrolysis/pyrogasification or catalytic (thermo)reforming. The following physicochemical features for the different biochar batches were measured: pH, conductivity, bulk density, humidity and ash content, particle size, total organic substances, and trace element concentrations. Fine porous structure analysis and total elemental analysis were performed using environmental scanning electron microscopy along with energy-dispersive X-ray spectrometry (EDX). Phytotoxicity tests were performed for each biochar. Finally, we were able to (i) differentiate the biochars according to their physicochemical properties, microstructure, elemental contents, and original raw biomass; (ii) correlate the whole biochar features with their respective optimal concentrations when used as plant fertilizers or soil improvers; and (iii) show that biochars from animal origin were phytotoxic at lower concentrations than those from plant feedstock.

## Introduction

According to the International Biochar Initiative (IBI) guidelines, “biochar is a solid material obtained from the thermochemical conversion of biomass in an oxygen-limited environment” (International Biochar Initiative [IBI], 2012). Charcoal is a carbon-rich solid product prepared via biomass pyrolysis and is used as a fuel source for producing energy. Charcoal and biochar constitute part of the black carbon by-products resulting from the combustion of organic matter (OM) that includes coal, soot, and graphite (Crombie et al., 2013). Biochar is currently a by-product of the transformation of biomass into bioenergy in thermochemical processes (Qambrani et al., 2017). The physicochemical properties of biochar are mainly connected to its large surface area, ranging from 200 to 400 m^2^ g^-1^. The thermal decomposition of organic material leads to loss of volatile compounds, producing a network of carbon chains present as porous structures with a large inner surface. In turn, this affects the retention of water, the capacity for binding metals and other elements and hosting microbial communities, and changes in soil structure such as porosity and density (Beesley et al., 2013; Joseph et al., 2013; Fellet et al., 2014). Biochar therefore possesses intrinsic capabilities for stimulating plant growth and increasing tolerance to abiotic stresses, by improving the water holding capacity of soils and nutrient retention because of its chemical and electrical properties (Jeffery et al., 2011).

A recent (2015) Italian law allows the use of biochar from plant biomass as a soil amendment and should favor the market introduction of biochars because of their greater efficiency and sustainability when compared to fossil carbon-based solutions. The application of biochar in agriculture would increase carbon storage in soils, provide cobenefits in saving water, avoiding emissions of greenhouse gasses from soils, substituting for fossil fuels used in the production of other soil-improving agents, and providing additional ecosystem services (Woolf et al., 2010). A detailed analysis of biochar benefits is reported by Schröder et al. (2018), in comparison with other types of soil-amending agents.

However, the benefits derived from biochar are strictly dependent on the original biomass (feedstock), on the production process, especially temperature, and on the characteristics affecting its performance as a soil improver (Mimmo et al., 2014; Agegnehu et al., 2017). Converting biomass into biofuels through thermochemical processes is one of the methods more accepted by both legislation and industry to reduce waste and obtain valuable products (Dodds and Gross, 2007). Catalytic (thermo)reforming and pyrolysis processes generate energy, bio-oil, gas, and biochar. In the former, a thermochemical process, catalyzed by specific elements/compounds transforms organic biomass into energy, gas, bio-oil, and a minimal amount of biochar. In this type of process, biochar can also be used as catalyst (Shen, 2015; Ahmad et al., 2018). Pyrolysis is defined as the thermal biomass decomposition under limited O_2_ and at an intermediate temperature to obtain energy, gasses, bio-oil, and biochar (Liu W.J. et al., 2017). The quality and elemental content of the biochar obtained during the two processes depends on the operating parameters, temperature, heating rate, type of catalyst, and on the physicochemical features of the feedstock.

New approaches to biochar production are under study in several research projects aiming at developing technologies where small quantities of pyrogases can provide heat to the process, and the remainder is available to supply further renewable heat for agricultural or industrial uses, improving the final energy efficiency and economy.

It has been calculated that globally 140 billion tons of biomass waste from agriculture are produced annually (United Nations Environment Program^1^, accessed March, 2018). In the European Union, feedstocks available from renewable sources consist mostly of residues from forestry and agriculture. Forestry residues can be primary, from the field, secondary, from the wood industry, or tertiary, from post-consumer wood. Recent data on biomass availability estimate a total of about 9 and 85 Mt of dry matter from forestry and agriculture respectively, not used for other purposes (Searle and Malins, 2016), that could be available for transformation into biochar applicable to agriculture. According to the EU Waste Framework Directive, the approach to waste management in EU Member States should be based on three main principles, listed in order of priority: (i) preventing waste, (ii) recycling and reusing, and (iii) improving final disposal and monitoring. The use of forestry and other plant maintenance residues to produce biochar is a positive step in activities aiming at producing environmental and economic advantages by upgrading waste material.

In this work, we compare biochars derived from different feedstocks (animal and plant) as sustainable products from waste management, with the aim to establish critical factors that affect the usefulness of biochars as soil improvers and amendments in agriculture, according to international guidelines. Some of the biochars’ chemical and physical characteristics were measured; phytotoxicity tests were carried out to establish for the different biochars the germination index using *Lepidium sativum* L. For two biochars, growth inhibition tests were performed using *Hordeum vulgare* L. The main findings were that the feedstock type determined the biochar microstructure and elemental composition, which were linked to the toxicity: biochar from animal origin were phytotoxic at lower concentrations than those from plant feedstock.

## Materials and Methods

### Biochar Origin and Production

Five different biochars were used in the study. Chars A1, A2, and A3 were obtained as by-products of catalytic (thermo)reforming in a prototype developed in the framework of the research project TERMOREF (Regione Emilia-Romagna); temperature was between 400 and 500°C for 2–3 h. Char A1 was derived from anaerobic digestate, char A2 from poultry litter, and char A3 from wood pellets. Char A4 was derived from wood pellets in a prototype pyrogasification system <50 kWe in the framework of the research project TERMOREF; temperature was between 500 and 700°C for 1–2 h. Char E1 was acquired from commercial sources and derived from forest wood and brushwood waste through pyrogasification (Borgo Val di Taro, Italy).

### Physicochemical Analyses

#### Sample Pretreatment

Biochar samples were sieved and prepared according to the protocol given in EN European Standards (2008).

#### pH Evaluation

The pH values of the biochar samples were determined following the guidelines of EN European Standards (1999a) protocol for growing media and soil improvers.

Briefly, samples were passed through a 20 mm sieve and extracted with deionized water in a ratio of 1:5 (v/v). To a sample weight equivalent of 60 mL, 300 mL of deionized water were added in 500 mL glass jars, and samples were shaken for 1 h on an orbital shaker (Model Unimax 2010, Heidolph Instruments, Schwabach, Germany). The pH of each suspension was measured in triplicate using a Model S213 Seven Compact Duo meter (Mettler Toledo, Columbus, OH, United States).

#### Electrical Conductivity Evaluation

Electrical conductivity (EC) values of the biochar sample extracts were determined following the EN European Standards (1999b) protocol for growing media and soil improvers.

Briefly, samples were treated as for the pH evaluation; after shaking, suspensions were filtered through Whatman filters (N°1, Whatman, Maidstone, United Kingdom) discarding the first 10 mL. The conductivities of the suspensions were measured, in triplicate, within an hour after extraction, using a Model S213 Seven Compact Duo meter (Mettler Toledo, Columbus, OH, United States), and expressed in mS cm^-1^.

#### Bulk Density Evaluation

The bulk density of the biochar samples were evaluated following the EN European Standards (2008) protocol, with minor modifications.

A rigid cylinder with a capacity of 1,000 mL and diameter of 100 mm was used to perform the experiment. The cylinder was filled with each sample and a plunger of 650 g with the same diameter of the cylinder was placed on the top for 3 min. After the compaction time, the plunger was removed and the sample weight and volume measured. Results were expressed in g L^-1^.

#### Particles’ Size Distribution Evaluation

The size distribution of the particles of the biochar samples was assessed following the EN European Standards (2007) protocol, with minor modifications.

Briefly, dried samples of biochar were passed through five sieves with different mesh sizes (20, 10, 5, 2, and 1 mm) positioned in tiers (Endecotts, Ltd., London, United Kingdom) from the largest to the smallest. A known mass of sample was placed on the upper sieve and the column was gently shaken by hand for 5 min. The biochar fractions retained on each sieve were then carefully collected and weighed, and the percentage fraction distributions calculated.

#### Dry Matter and Moisture Content Measurements

Dry matter and moisture content of the biochar samples were determined following the EN European Standards (2008) protocol.

Briefly, samples of known weight were oven-dried at 105°C (M710 Thermostatic Oven, F.lli Galli, Milan, Italy) until the difference between two successive weightings did not exceed 0.1 g. The final weight of the samples represents the dry matter content, and the weight loss represents the moisture content; moisture content is expressed as a percentage of the starting weight.

#### OM and Ash Content Evaluation

Organic matter and ash content of the biochar samples were determined according to the EN European Standards (2011) protocol.

Fresh samples were oven-dried at 105°C (M710 Thermostatic Oven, F.lli Galli, Milan, Italy) to a constant weight, and a known weight of the sample was incinerated at 500°C in a muffle furnace (Model A022, Matest S.p.A., Bergamo, Italy) for 14 h. After incineration, the residues (ash) were weighed and OM was calculated as the difference between the fresh and final incinerated weights. Ash content and OM content were expressed as a percentage of the total initial weight.

#### Measurement of Trace Metal Concentrations: Cu, Fe, Ni, Zn, Pb, and Cd

The concentration of six trace metals (Cu, Fe, Zn, Pb, Ni, and Cd) in the biochar samples were determined following the UNI Ente Italiano di Normazione (1998) protocol, with minor modifications.

Samples were oven-dried at 105°C (M710 Thermostatic Oven, F.lli Galli, Milan, Italy) up to a constant weight, and a known weight of the sample was incinerated at 500°C in a muffle furnace (Model A022, Matest S.p.A., Bergamo, Italy) inside ceramic crucibles with lids for 14 h. For each biochar, the ash was retrieved from the crucible and solubilized by wet digestion with HNO_3_ 65% (Carlo Erba, Milan, Italy) at 165°C for 30 min and 230°C for 30 min in a heated digester thermoblock (DK20, Velp Scientifica, Usmate Velate, MB, Italy). Digested solutions were diluted with deionized water to 30% (v/v) acid concentration. The concentration of each metal was measured using flame atomic absorption spectrometry (AA240FS, Agilent Technologies, Santa Clara, CA, United States) at the following wavelengths: λ Cu: 324.7 nm; λ Fe: 248.3 nm; λ Zn: 213.9 nm; λ Pb: 217.0 nm; λ Ni: 232.0 nm; λ Cd: 228.8 nm. Calibration curves for each metal were prepared using 1,000 ppm certified standard solutions (Agilent Technologies, Santa Clara, CA, United States). Three instrumental replicates were performed on each biological replicate (three for each sample). The metal concentrations were expressed in mg kg^-1^ of biochar.

#### Low-Vacuum Scanning Electron Microscope With X-Ray Microanalysis (Lv SEM/EDX)

To overcome the problems with high vacuum, an environmental low-vacuum (Lo-vac, 60 Pa) environmental scanning electron microscopy (ESEM/EDX) was used. Finely-powdered biochar (between 10 and 100 μm grain size) was analyzed with no fixation or staining, after a careful positioning and adhesion on 2 cm diameter stainless-steel sample holders covered with adhesive carbon tape. A scanning microscope ESEM FEG2500 FEI (FEI Europe, Eindhoven, Netherlands), operating in low-vacuum (70 Pa) with a large-field detector allowed optimal secondary electron imaging, while the cone pressure-limiting aperture set at 500 μm improved the signal available to the Bruker XFlash^®^6 | 30 X-ray detector equipped with a high efficiency 30 mm^^2^^ silicon drift detector for nanoanalysis and high-count-rate spectral imaging (Bruker Nano GmbH, Berlin, Germany). Secondary electron imaging was performed at 5 or 10 keV with a beam size of 2.5 μm, and EDX analysis at 20 keV acceleration voltage and beam size of 4 μm. The working distance was approximately 10 mm, and the scanning time 1–3 μs. The xT Microscope Control, xT Microscope Server, and FEI User Management software were used for imaging; the Esprit 1.9 package was used for X-ray spectra acquisition and analysis during acquisition in either point analysis or line-scan mode. X-ray spectra deconvolution and elemental standardless quantification were performed using the P/B-ZAF interactive method [Peak/Background evaluation matrix with atomic number (Z), absorption (A), and secondary fluorescence (F) correction] supported by the “Quantify Method Editor” option of Esprit 1.9 (Goldstein et al., 2003).

### Effects on Growth and Germination

#### Germination Test

The germination index of the biochar matrices was determined following the UNICHIM (2003) protocol.

Biochar samples were sieved to pass a 2 mm mesh. Aliquots of 10, 7.5, 5, 2.5, 1, 0.5, or 0.1 g of dried and sieved biochar were placed in Petri dishes (Ø 90 mm, Sarstedt, Germany) and saturated with deionized water. The suspension was homogenized and covered with a Whatman N°41 filter (Whatman, Maidstone, United Kingdom). Control dishes were prepared containing only deionized water and filter paper. Ten seeds of *Lepidium sativum* L. (Sementi Dotto Spa, Udine, Italy) were sown in every Petri dish; three biological replicates were performed for each concentration. Sealed dishes were placed in a growth chamber (MIR-554-PE, Panasonic, Osaka, Japan) at 25°C in the dark. After 72 h, the germinated seeds were counted, and their root lengths were measured to calculate the germination index (GI %).

GI % = (Gt ^∗^ Lt/Gc ^∗^ Lc) ^∗^ 100.

where Gc = germinated seeds in the control; Gt = germinated seeds in the treatments; Lc = main root length in the control; Lt = main root length in the treatments.

#### Germination Rate and Growth Inhibition Tests

Germination and growth inhibition tests were performed following the EN European Standards (2012) protocol.

The growing substrates were prepared according to the protocol by mixing char at doses of 1, 3, or 5% (w/v) with sphagnum peat (Lithuanian peat, UAB Presto Durpes, Vilnius, Lithuania) limed at pH 5.5–6 with CaCO_3_ (Sigma-Aldrich, St. Louis, MO, United States), using commercial garden soil as a control (Ecomix, Vialca S.r.l., Pistoia, Italy). Peat had an EC of 400 μS cm^-1^ and a starting pH of 5.1, and concentration of all metals were below detection limits. Approximately 700 mL of the different mixtures of peat and biochar were distributed in 1,500 mL glass jars and fertilized initially with half-strength Hoagland solution (J.T. Baker, Deventer, Holland) at the rate of 40 mL per kilogram of substrate; each treatment was repeated in triplicate. Control pots were prepared without biochar. Twenty seeds of *Hordeum vulgare* L. (provided by CIEMAT, Madrid, Spain) were sown in each jar. Jars were kept in a greenhouse under controlled conditions: average temperature of 27°C during day-time, 16°C during night-time, with natural light supplemented by metal halide lamps to maintain a minimum light intensity of 300 μmol m^-2^ s^-1^ and a photoperiod of 14 h. Jars were watered daily with 10 mL of deionized water. After 5 days, the number of germinated seeds was recorded to calculate the germination rate percentage:

Germination rate % = (number of germinated seeds/number of starting seeds) ^∗^ 100.

After 11–14 days, when the second true leaf was clearly visible in 50% of the plants in control condition, the above-ground parts of all plants were collected, thoroughly washed, and weighed. The material was then oven-dried at 75°C to constant weight. The relative growth index (RGI) for each biochar type and growing medium concentration was calculated for both fresh and dry plant biomass:

(RGI) F/D = (Wt/Wc)F/D

where Wt = fresh or dry weight of treated plants; Wc = fresh or dry weight of control plants.

### Statistical Analyses

All statistics was calculated using IBM-SPSS v25^2^. After verifying the normal distribution and the homogeneity of variance with Levene’s test, an analysis of variance (ANOVA) was carried out on the data, followed by Tukey’s *post hoc* test. Dimension reduction calculations were applied to the physicochemical analyses: principal component analysis (PCA) extraction with the Kaiser criterion (λ > 1) and canonical discriminant functions analysis. For testing phytotoxicity, Student’s *t*-test with the Bonferroni correction was used.

## Results

### Comparison of Physicochemical Features in Different Chars

**FIGURE 1:**
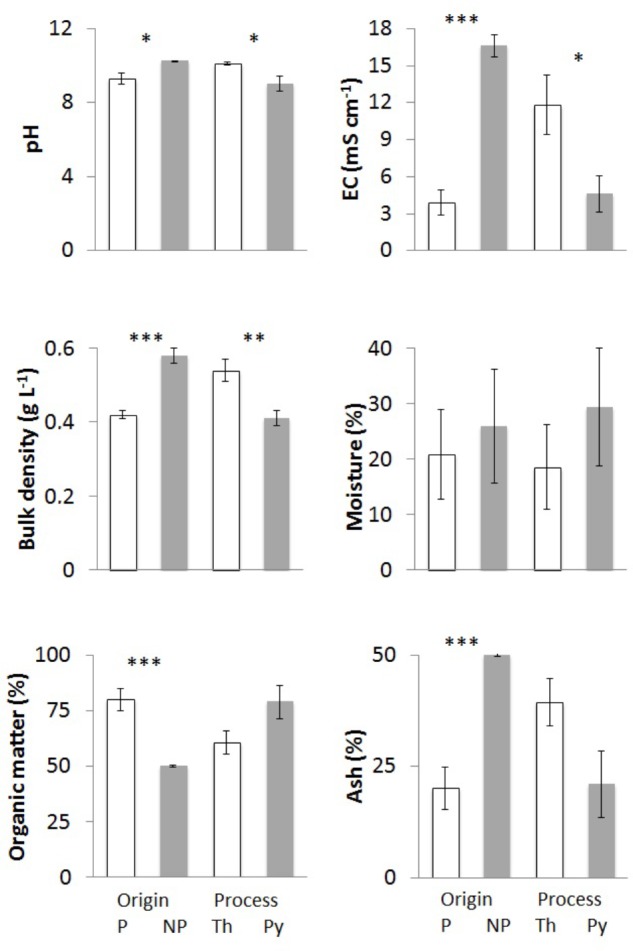
Physicochemical parameters of chars from different feedstock and production process: pH, electrical conductivity, bulk density, moisture, organic matter, and ash content. Bars “P” indicate chars of plant origin (A3, A4, E1 – white) and bars “NP” chars of non-plant origin (A1, A2 – gray). Bars “Th” indicate chars from (thermo)reforming (A1, A2, A3 – white), and “Py” chars from pyrolysis (A4, E1 – gray). Means of at least three biological replicates per char are reported, with standard error of the mean. Asterisks show the significance of pairwise comparisons (one-way ANOVA) between chars derived from different feedstocks (P/NP) or from different processes (Th/Py): ^∗^*p* < 0.05; ^∗∗^*p* < 0.01; ^∗∗∗^*p* < 0.001.

**FIGURE 2:**
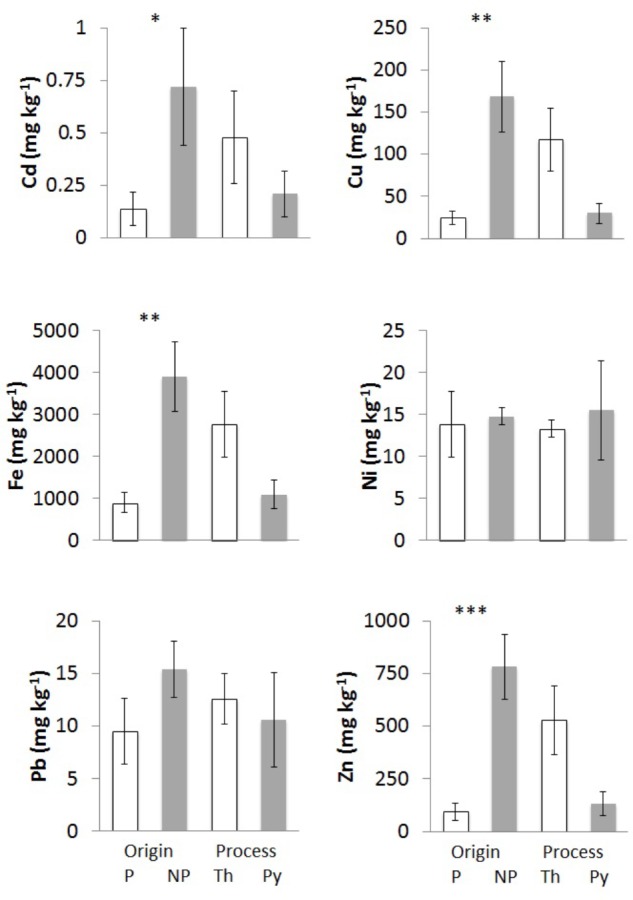
Trace element concentrations of chars from different feedstocks and production processes: Cd, Cu, Fe, Ni, Pb, and Zn. Bars “P” indicate chars of plant origin (A3, A4, E – white) and bars “NP” chars of non-plant origin (A1, A2 – gray). Bars “Th” indicate chars from (thermo)reforming (A1, A2, A3 – white), and “Py” chars from pyrolysis (A4, E1 – gray). Means of at least three biological replicates per char are reported, with standard error of the mean. Asterisks show the significance of pairwise comparisons (one-way ANOVA) between chars derived from different feedstocks (P/NP) or from different processes (Th/Py): ^∗^*p* < 0.05; ^∗∗^*p* < 0.01; ^∗∗∗^*p* < 0.001.

The applicability in agriculture of chars for soil amendment is strongly affected by their characteristics including structure, chemical composition, and levels of contaminants. Several studies on the standardization of measurements and the choice of relevant parameters are underway (Bachmann et al., 2016). The chars analyzed in the present study share the same production family of origin because they are all surplus agricultural waste; their reuse follows the principles of zero residues within the framework of a circular economy approach (Riding et al., 2015). Three chars (A3, A4, E1) were produced from wood, either pelleted or chipped, whereas two chars (A1, A2) were produced from non-plant sources, digestate and poultry litter respectively. The processes used in producing chars were different, using prototypes for (thermo)reforming and pyrogasification (A1-A2-A3 and A4-E1 respectively). In view of these differences, their physicochemical parameters (**Tables 1–3**) were analyzed to provide information about possible correlations between biomass of origin, production process, and char features (**Figures 1**, **2**).

**Table 1:** Physicochemical characterization of chars from thermocatalytic reforming of digestate (A1), poultry litter (A2), and wood pellet (A3) and from pyrogasification of wood pellet (A4) and brushwood (E1).

Parameter	A1	A2	A3	A4	E1
pH	**10.23** (0.01) a	**10.25** (0.04) a	9.84 (0.03) b	8.11 (0.04) c	9.93 (0.04) b
Electrical conductivity (mS cm^-1^)	18.6 (0.2) a	14.5 (0.2) b	2.4 (0.1) d	1.4 (0.1) e	7.9 (0.1) c
Bulk density (g cm^-3^)	0.54 (0.01) b	0.62 (0.02) a	0.45 (0.02)c	0.37 (0.01) d	0.44 (0.01) c
Moisture content (% fresh weight)	2.91 (0.02) b	48.88 (0.64) a	3.73 (0.40) b	6.29 (0.30) b	52.46 (5.11) a
Organic matter (% dry weight)	50.57 (0.25) d	49.42 (0.32) d	81.89 (0.32) b	95.63 (0.12) a	62.32 (0.33) c
Ash (% dry weight)	49.4 (0.3) a	50.6 (0.3) a	18.1 (0.3) c	4.4 (0.1) d	37.7 (0.3) b

*Means of three biological replicates are reported with standard errors in parentheses. Numbers in bold type represent values that exceed the prescriptions of international guidelines (REFERTIL Consortium, http://www.refertil.info/) on soil improvers. Different lower case letters in rows indicate the significant difference at *p* < 0.05 (Tukey HSD test)*.

**Table 2:** Particle size distribution of chars from thermocatalytic reforming of digestate (A1), poultry litter (A2), and wood pellet (A3) and from pyrogasification of wood pellet (A4) and brushwood (E1).

Particle size	A1	A2	A3	A4	E1
10 > x > 5	58.7	3.7	3.0	8.4	0
5 > x > 2	40.3	10.4	36.3	61.7	4.0
2 > x > 1	0.3	15.0	11.7	10.4	10.0
<1	0.7	**70.8**	**49.0**	**19.5**	**86.0**

*Data are expressed as the percentage of the total weight of three replicates. Numbers in bold type represent values that exceed the prescriptions of international guidelines (REFERTIL Consortium, http://www.refertil.info/) on soil improvers*.

**Table 3:** Total concentration of trace elements in chars from thermocatalytic reforming of digestate (A1), poultry litter (A2), and wood pellet (A3) and from pyrogasification of wood pellet (A4) and brushwood (E1).

Trace element	A1	A2	A3	A4	E1
Cd	0.09 (0.02) c	**1.35** (0.06) a	<0.001	<0.001	0.42 (0.11) b
Cu	73.91 (1.12) b	**262.07** (8.36) a	14.40 (0.92) c	2.91 (0.04)c	56.74 (1.39) b
Fe	5735 (126) a	2060 (5) b	505 (18) c	340 (13) c	1839 (151) b
Ni	13.47 (1.20) bc	16.20 (1.23) b	10.35 (0.60) c	2.23 (0.03) d	28.75 (0.67) a
Pb	12.4 (5.2) ab	18.3 (0.8) a	7.2 (3.4) ab	1.6 (0.1) b	19.6 (4.3) a
Zn	435.3 (7.2) b	**1128.7** (11.0) a	18.2 (1.6) d	4.5 (0.2) d	260.6 (9.2) c

*Means in mg kg^-*1*^ dry weight of three biological replicates are reported with standard errors in parentheses. Numbers in bold type represent values that exceed the prescriptions of international guidelines (REFERTIL Consortium, http://www.refertil.info/) on soil improvers. Different lower case letters in rows indicate the significant difference at *p* < 0.05 (Tukey HSD test)*.

#### pH and Electrical Conductivity

All chars showed high pH values greater than 8 (**Table 1**). There were significant differences, and the highest values were found in the chars of non-plant origin (**Figure 1**). Compared to (thermo)reforming, pyrogasification produced chars with significantly lower pH (**Figure 1**).

Electrical conductivity values were extremely variable (**Table 1**), with highly significant differences linked to the origin of the biomass (**Figure 1**); chars derived from wood showed low values of conductivity compared to chars of non-plant origin, with fourfold differences.

#### Density and Particle Size Distribution

Densities of the chars ranged around 0.5 g L^-1^ (**Table 1**), with the highest values found in chars of non-plant origin produced via (thermo)reforming (**Figure 1**). The distribution of particle size, determined through sequential sieving, did not correlate strictly with density (**Table 2**). Char E1, from chipped brushwood, had the highest proportion of very fine particles of less than 1 mm diameter. A similar size distribution was found in char A2 from poultry litter. Char A1 from digestate had the highest proportion of large particles, mostly of size greater than 5 mm. In char A3, produced by pyrogasification from wood pellet, most particles were of size between 2 and 5 mm. The size of particles is a critical factor in their distribution and applicability in agricultural conditions, since fine char particles cannot be distributed easily except after transformation into sludge.

#### Moisture Content, Organic Matter, and Ash

After the production process, char might contain considerable amounts of water. Moisture content of the original biomass is one of the most critical features for the feed-in process and the efficacy of combustion. The proportion of moisture in fresh chars, which is complementary to the dry weight of the char, is shown in **Table 1**. There is no correlation with the type of biomass or the type of process (**Figure 1**), and the highest values for moisture are found in poultry litter char A2 and in brushwood char E1.

The dry matter in char can be further divided into OM and residual ash after incineration at 500°C. **Table 1** reports both values that together equal 100% of dry weight. Significant differences are evident, with chars from wood having significantly higher OM content and less ash (**Figure 1**). No effect can be linked to the production process.

#### Content of Trace Metals

One of the potential hazardous features of chars is the possible increase in concentrations of metals and other contaminants due to the reduction of volume and water content from the original biomass. It is expected that all non-volatile elements become more concentrated in char at increasing temperatures. The concentrations on a dry weight basis of six main trace elements were determined and results are reported in **Table 3**. The elements Cu, Fe, and Zn, which are micronutrients, could originate from the biomass of origin, being essential constituents of cells, whereas Cd, Ni, and Pb should be present only as contaminants.

Iron is present in the chars at the level of g kg^-1^ dry weight (**Table 3**), with the highest values in chars of non-plant origin (**Figure 2**). Char E1 is an exception since its Fe content is much higher than in chars derived from wood pellets; a possible explanation lies in the origin of the wood as waste in forests and undergrowth, possibly mixed with small amounts of soil and of materials of diverse origin. Chars from pure wood, A3 and A4, have the lowest Fe levels.

Zinc and Cu also reach high levels in chars of non-plant origin (**Table 3**), with highly significant differences (**Figure 2**). Differences in concentrations of Cu and Zn of >100-fold are evident comparing poultry litter char A2 and wood char A4.

Nickel and Pb concentrations do not show any significant differences among chars of different origin or production process (**Figure 2**), but the values were highly variable.

Cadmium was below the detection limit in chars from pure wood pellet, independent of the production process (**Table 3**), while values were significantly higher in chars of non-plant origin (**Figure 2**).

#### Classification of Chars Based on Physicochemical Properties

**FIGURE 3:**
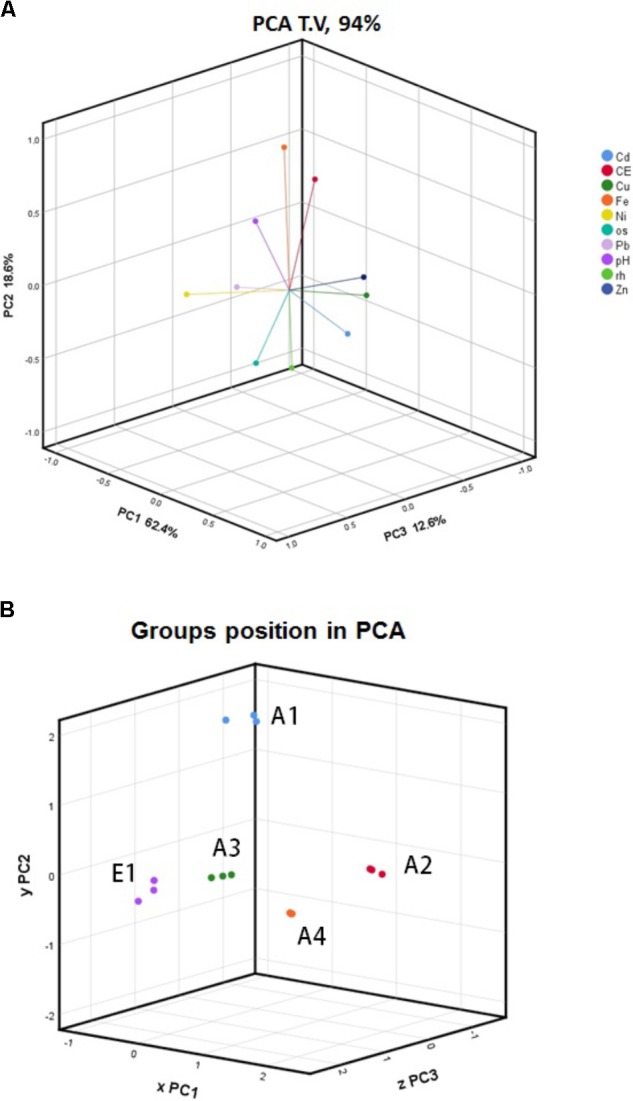
Principal component analysis (PCA) of **(A)** physicochemical parameters of chars and **(B)** char data sets. All data gathered from all biological replicates of five chars, A1, A2, A3, A4, and E1, were analyzed using PCA. The legend indicates the color codes of the different parameters: CE, electrical conductivity; os, organic matter; rh, moisture content.

The complete set of data from all replicates and for all parameters was subjected to PCA. In this analysis, OM was retained instead of ash content, and moisture content was retained instead of dry matter; bulk density and particle size distribution were not considered. **Figure 3A** shows the trends of the different parameters as vectors in three dimensions accounting for 93.6% of variance (PC1 = 62.4%, PC2 = 18.6%, PC3 = 12.6%). In the first two dimensions (**Supplementary Figure 1a**), OM content is clearly separated from all other parameters; Cu, Cd, and Zn group together; and pH parallels EC. The second and third dimensions discriminate pH from conductivity and Fe from Ni. Considering the vector lengths, which indicate their relevance in explaining the total variance, and their loading coefficients along the principal components (**Supplementary Figures 1b–d**), we can deduce the best variables for discriminating among chars of different origins and processes. Among the metals the best variables are Cu (PC1), Fe (PC2), and Ni (PC3), and among the other parameters EC (PC2) and OM (PC1 and PC3). Using a 3D representation of the datasets (**Figure 3B**), it can be seen that chars A3, A4, and E1 were grouped together while A1 and A2 were separate but independent of one another.

**FIGURE 4:**
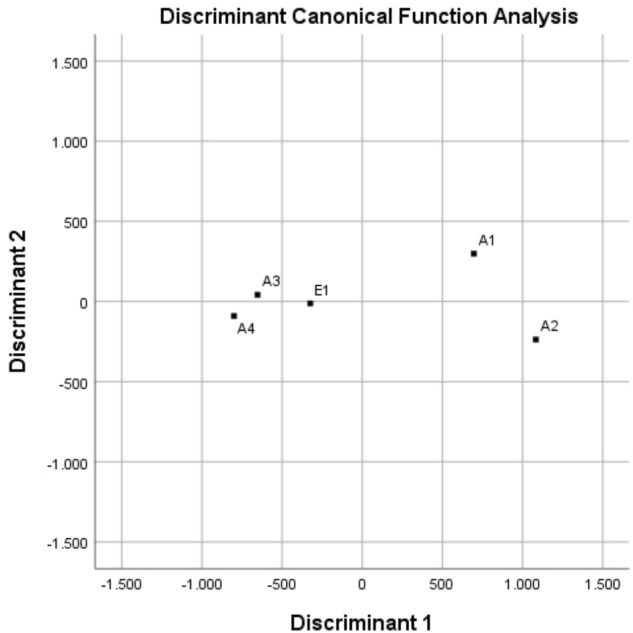
Discriminant canonical function analysis of data sets for chars A1, A2, A3, A4, and E1, derived from different feedstocks and processes.

Through the canonical discriminant function analysis, it was possible to divide the whole variance between only two canonical (orthogonal) functions. This technique, which is a simplified PCA, allowed grouping the different chars in a 2D graph, as shown in **Figure 4**. Chars from wood—A3, A4, and E1—group together and quite distinctly from the chars of non-plant origin—A1 and A2—that do not cluster together. There is no clustering associated with the production process.

### Microstructural Features of Chars

**FIGURE 5:**
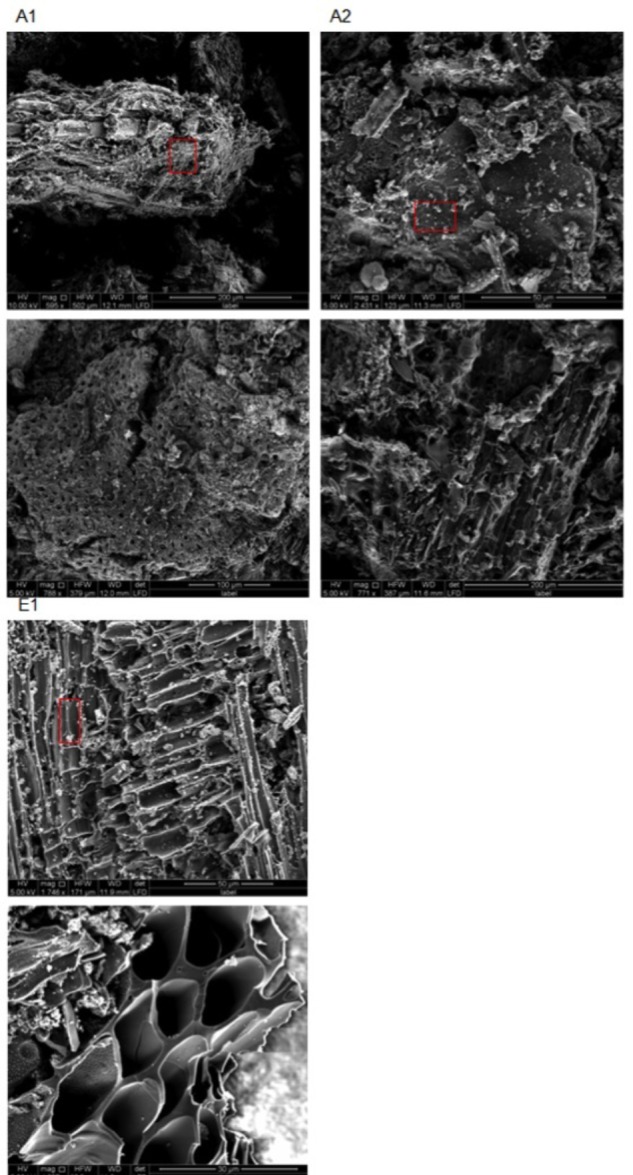
Environmental scanning electron microscopy (ESEM) images of biochars A1 (2 top left), A2 (2 top right), and E1 (2 bottom left) at different magnifications (see figure bottom and white bar bottom right). All images were acquired with electron beam at 5 KV and a working distance between 11.9 and 12 mm. Red rectangles indicate the areas from which the EDX spectra in **Figure 7** were acquired.

**FIGURE 6:**
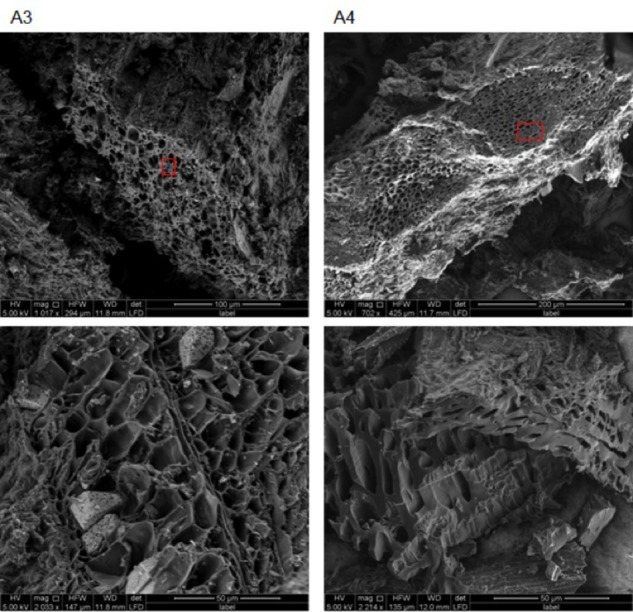
Environmental scanning electron microscopy images of biochars A3 (2 left) and A4 (2 right) at different magnifications (see figure bottom and white bar bottom right). All images were acquired with electron beam at 5 KV and a working distance between 11.9 and 12 mm. Red rectangles indicate the areas from which the EDX spectra in **Figure 8** were acquired.

SEM images obtained for chars A1, A2, and E1 demonstrate the presence of plant and non-plant matrices (**Figure 5**). Chars A1 and A2, from digestate and poultry litter respectively, showed mostly animal substrates that appeared as lumps encrusted with fine and coarse powder (**Supplementary Figure 2**). In the case of char E1, we observed a prevalence of highly porous matrix, which was interspersed with lumpy structures covered with micrometer-sized powder (**Figure 5** and **Supplementary Figure 3**). Exclusively porous substrates were seen in chars A3 and A4 (**Figure 6**). In particular, in A3 we observed pores of different sizes, between 5–20 μm and larger than 20 μm, while in A4 the pores were almost exclusively between 5 and 20 μm. Both the porous matrices appeared disorganized with pores of different sizes distributed randomly (**Figure 6** and **Supplementary Figure 4**).

**FIGURE 7:**
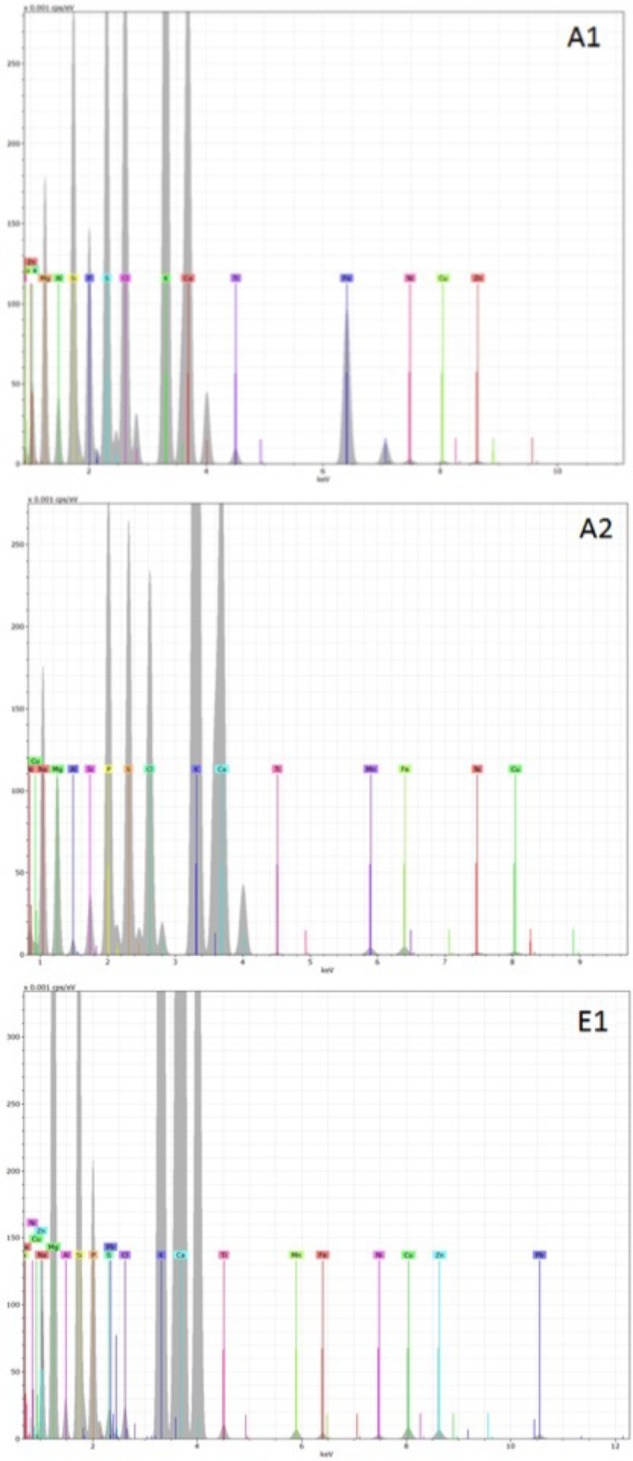
Deconvoluted X-ray emission spectra acquired from biochars A1, A2, and E1. The electron beam energy was set at 20 KV and the acquisition live time at 60 s. In each spectrum, the X axis represents the X-ray energy in eV, and the Y axis represents the peak intensity.

**FIGURE 8:**
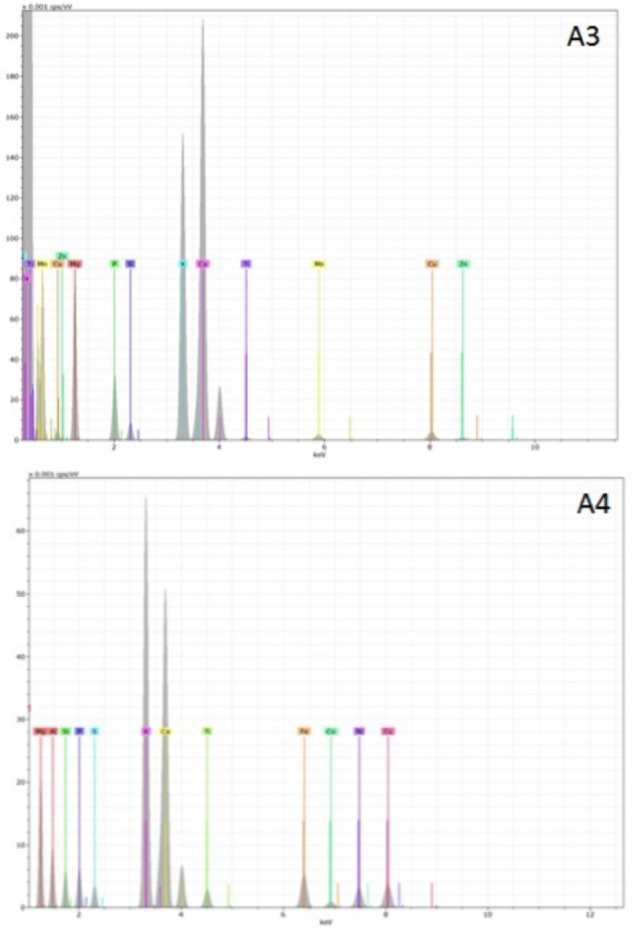
Deconvoluted X-ray emission spectra acquired from biochars A3 and A4. The electron beam energy was set at 20 KV, and the acquisition live time at 60 s. In each spectrum, the X axis represents the X-ray energy in eV, and the Y axis represents the peak intensity.

Representative examples of electron-induced emitted X-ray spectra for the different char types are reported in **Figures 7**, **8**. On average, 25 X-ray spectra were collected for each biochar to gather a reliable set of measurements of their elemental content. For all chars, the X-ray analysis confirmed the presence of Fe, Cu, Zn, Ni, and Pb, but other metals were also found, for example Ti and Mn. Concentrations of Cd were below the detection limit for all samples. Spectra for chars A1 and E1 confirm the high content of Fe. Chars differed in their contents of light elements as well. Chars A1, A2, and E1 were rich in Si, K, and Ca while in particular the chars of non-plant origin were rich in S, Na, Cl, P, and Mg (**Figures 7**, **8**). Chars A3 and A4 from pure wood pellets showed lower amounts of all light elements and metals in comparison to the other biochars (**Figure 8**). The contents of light elements were comparable in A3 and A4, but K and Ca were lower in A4 than in A3 (**Figure 8**).

### Effect of Chars on Germination and Growth of Plants

#### Germination Index

**FIGURE 9:**
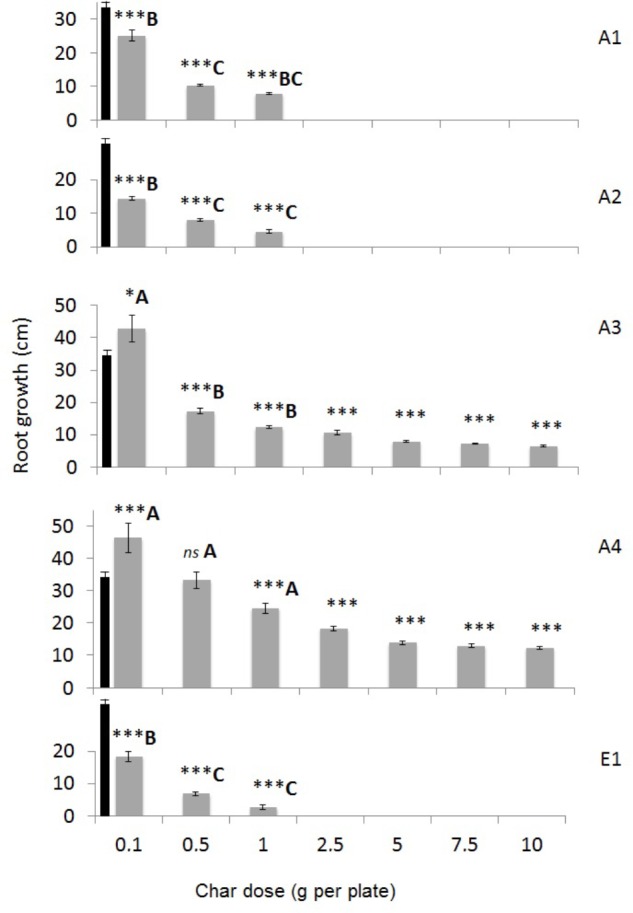
Root growth of *Lepidium sativum* seedlings germinated for 72 h in the presence of chars from different feedstocks and production processes at increasing concentrations (0.1 to 10 g per plate). Means of 30 seeds are reported with standard errors. Growth in distilled water (control) was 34.29 ± 1.31 cm and is shown as a black bar in all graphs. Asterisks indicate significant differences in the growth of treated samples with respect to control: ^∗^*p* < 0.05; ^∗∗∗^*p* < 0.001. Capital letters indicate significant differences assessed with one-way ANOVA at *p* < 0.05 between chars tested at the same concentration (in the conditions 0.1, 0.5, and 1 g per plate).

A basic phytotoxicity test was performed by using germinating seeds of *Lepidium sativum* on Whatman paper in the presence of increasing quantities of char in water. The effect is measured by taking into account the number of seeds that germinate and the growth of roots after 3 days of incubation, compared to the control with water (**Figure 9** and **Supplementary Figure 5**). Chars A3 and A4 from pure wood pellets, irrespective of the production process, stimulated plant germination and root growth at the lowest dose, 0.1 g per plate. Char A4 was significantly less toxic than A3 at the higher doses, with a germination index greater than 20%. On the contrary, chars A1, A2, and E1 were strongly inhibitory, and stopped growth altogether at doses between 1 and 3 g per plate. Considering only the germinated seeds, **Figure 7** shows the differences in root growth at different doses, demonstrating the positive effects of chars A3 and A4 and the inhibitory effects of chars A1, A2, and E1.

#### Relative Growth Index

**FIGURE 10:**
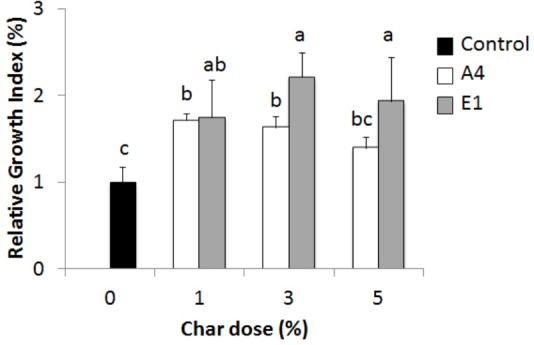
Relative growth index (RGI) of *Hordeum vulgare* seedlings germinated for 72 h in the presence of chars from pyrolysis of different feedstocks at increasing concentrations (1 to 5%). White bars, char A4; gray bars, char E1. Means of 30 seeds relative to growth of the control in pure soil (black bar) are reported with standard errors. Letters indicate significant differences assessed with one-way ANOVA at *p* < 0.05.

Evaluation of the effects of chars on plant growth can also be assessed in pot experiments, by measuring a RGI at different concentrations of char mixed into the growth substrate (soil or peat). The test was performed on *Hordeum vulgare*, comparing the best- (A4) and worst- (E1) performing chars according to the germination index (**Figure 10**). Char A4 from pyrolysis of pure wood pellets significantly enhanced plant growth at the concentrations of 1, 3, and 5%. Unexpectedly, char E1 also enhanced the growth of plants in comparison to control, with stimulating effects at 3%. In this test on barley, chars E1 and A4 were found to be non-phytotoxic and promising as soil amendments. The dose of 1% in pots corresponds to a distribution of about 45 t ha^-1^ of char in the field, assuming dispersal in the first 30 cm of topsoil.

## Discussion

The choice of feedstock in biochar production has a significant effect on the properties of the product, its applicability, the benefits in terms of reduction in greenhouse gas emissions, and economic advantages (Galinato et al., 2011; Field et al., 2013). In addition, using biochar as a way of disposing several types of agricultural and forestry wastes complies with the circular economy approach, advocated in several policies and strategies in the European Union and in other countries (Monlau et al., 2016). However, the evaluation of the benefits in adding biochar to agricultural soils has been so far inconclusive (Jeffery et al., 2011; Agegnehu et al., 2017), even though several studies suggest that biochar addition could provide considerable advantages, but also has a few drawbacks (Tan et al., 2017; Schröder et al., 2018). Characterization of the main properties of chars derived from different feedstocks and different production systems is therefore a necessity to identify the main parameters that can guide the decision process before a full-fledged application of biochar to agricultural crops (Tan et al., 2017).

The chars considered in our analysis were obtained from wastes from agriculture and forestry, and they were produced using two different processes at similar temperatures (around 500°C), (thermo)reforming and pyrolysis/pyrogasification. The properties of the chars were studied to understand the main factors in their application as soil improvers in agriculture.

The distribution of particle size is a relevant factor, since the finest particles can be released to the atmosphere during handling, consequently leading to an undesirable negative impact, thus counteracting the positive effects in terms of climate-altering emissions. In addition, fine particles in the soil decrease the water holding capacity compared to coarser particles (Liu Z. et al., 2017). Pelletizing chars could improve the handling procedures but on the other hand might decrease the large surface and microporosity, which are some of the main positive properties of char (Erlich et al., 2006; Karkania et al., 2012). Wetting biochar is probably a better solution to control small particles (Maienza et al., 2017), as is the case with some of the chars studied here.

The highest ash content in our study was found in chars derived from digestate and poultry litter, in line with the data on char from organic waste obtained through slow pyrolysis and other processes (Field et al., 2013; Qambrani et al., 2017). On the contrary, chars from wood have ash content <10%. Products with an ash content >50% are not compatible with the definition of biochar and are classified as pyrogenic carbonaceous material (European Biochar Certificate [EBC], 2015).

Microscopic examination of the different chars provided evidence of how the animal feedstocks, litter, and digestate became biochar matrix devoid of pores; conversely, plant substrates became porous biochar (Beesley and Marmiroli, 2011; Lu et al., 2012; Song et al., 2014). Biochar E1 showed a combination of characteristic wood-derived micro-, meso-, and macro-pores, covered with fine dust and interspersed with lumps of non-plant origin (**Supplementary Figures 3c,d**). In A3 and A4, the porous matrix appeared disorganized with pores of different sizes distributed at random. These structures could be ascribed to the wood pelleting process that brings together wood fragments with diverse structures (Karkania et al., 2012; Xue et al., 2013). The lower level of porosity of animal-derived chars, observed by other authors (Lu et al., 2012; Song et al., 2014), makes them less effective in retaining water in soil (Liu Z. et al., 2017).

Metals and trace elements were analyzed after wet digestion, and with X-ray analysis accompanying SEM. The data from EDX analysis were semi-quantitative, while those from the chemical analysis were quantitative. For many elements, such as Fe, Ni, Zn, and Cu, both techniques confirmed the elemental presence in all chars. EDX identified additional elements such as titanium and manganese, which might be derived from stainless steel components in the processing equipment (Fellet et al., 2014). The presence of heavy metals in high concentrations could be a factor in phytotoxicity (Jones and Quilliam, 2014), particularly evident in chars of non-plant origin analyzed in the present study. The possible release of toxic metals, present in the original feedstock and concentrated into the chars, has been considered a general hazard in the use of chars derived from animal waste, such as sludge and manure (Garlapalli et al., 2016; Wu et al., 2016). However, the high phytotoxicity of chars A1 and A2 could be explained in terms not only of metals, but also of excessive levels of S, Na, and Cl. For example, poultry litter feedstocks are normally rich in S, Na, and Cl and so are the chars obtained from them (Cantrell et al., 2012; Novak et al., 2013). In the case of sewage sludge, there is a wide variation in element content; however, S and Na are abundant and concentrated in derived biochars, as has been reported in other studies (Lu et al., 2012; Song et al., 2014; Mierzwa-Hersztek et al., 2017).

The toxicity of char E1 might be explained partly by its high Ni and Pb content, accompanied by a low pH (**Figure 7** and **Table 1**). A low pH is commonly associated with an increased concentration of aluminum (Al), which is highly toxic to plant roots (Gruba and Mulder, 2008). It is however interesting that while char E1 was toxic in germination tests on *Lepidium sativum*, it was highly phytostimulating in growth tests with *Hordeum vulgare*. Similar differences in the response of dicots and monocots have been demonstrated by Knox et al. (2018) using chars from wood pellets. It appears that the phytotoxic or phytostimulating activities of the biochar could be related not only to their metal content, but also to their macro- or micronutrient (K, Ca, S, P, Cl) content because it contributes to the salinity of the growth substrates (Farifteh et al., 2008). It has been shown that the high K content in many chars increases soil K availability and reduces K leaching (Laird et al., 2010). Phosphorus is often tightly bound in soils rich in Fe and Al oxides, but biochar addition increases soil pH, making P more available to plants and microorganisms (Asai et al., 2009; Hale et al., 2013).

It is known that carbonization processes can lead to phytotoxic products, such as PAHs or phenols, and other contaminants might be present depending on the input feedstocks used in the production of the chars (Bachmann et al., 2016). Organic compounds have not been analyzed in this study. Knox et al. (2018) attribute part of the phytotoxic effects to volatile compounds emitted by the biochar, and such emissions could be particularly important when tests are performed in closed containers, such as Petri dishes.

Restoring carbon in soils with biochar fits into the objectives of climate change priorities in Europe and in the world (Barrow, 2012). Biochar has also been proposed as a peat substitute, after the Commission Decisions of 2006 for soil improvers and for growing media specifying that the EU Ecolabel scheme for these products should apply only to those containing neither peat nor OM in their formulations. However, the application of biochar in real agricultural conditions requires simultaneous deployment of other improvers, such as OM, or standard fertilizers (Agegnehu et al., 2017; Bonanomi et al., 2017). It has been demonstrated recently that a coating of OM on the surface of biochar particles after co-composting increases the benefits to soil (Hagemann et al., 2017). In a recent experiment on arid and acidic Nepalese soils, wood biochar addition at 2% (v/v), implemented with NPK fertilization, significantly increased soil water retention capacity, plant-available water, increased soil EC, pH, exchangeable K^+^, Mg^2+^ and Ca^2+^, plant-available P, and organic C, giving rise to significantly improved maize biomass production in comparison to NPK fertilization (Pandit et al., 2018). However, the increase in soil EC corresponds to increase in salinity, which can lead to soil infertility (Farifteh et al., 2008). Therefore, careful land management should be followed according to the type of soil on which the biochars are applied: clay soils have a greater salinity buffer potential than sandy soils, and accurate balance between irrigation and drainage avoids the build-up of ions in the root zone, thus reducing exposition of plants to salt stress (Payen et al., 2016). Another study evaluated the application in a weathered tropical soil of biochar derived from sewage sludge, in combination with mineral fertilizer; after 2 years, only P availability from soil was increased by biochar, but overall the plant production rate increased (Monteiro Faria et al., 2017). These two experiments demonstrate that the direct effects of biochar on soil can be diverse, depending on the feedstock, on the overall biochar features, and on the soil type. However, accompanied by sensible land management, a predominantly stimulating effect on plant growth and yield was observed in both cases.

In this work, we were able to differentiate the biochars according to their physicochemical properties, microstructure, elemental contents, and the raw original biomass and to correlate these features with their respective optimal concentrations when used as plant fertilizers. In particular, biochars of animal origin were more phytotoxic at low concentrations than those of plant origin. In conclusion, it appears that additional experiments are needed to determine other characteristics of biochars to facilitate the use of appropriate feedstock and processing technologies to produce biochars that can be used as safe soil improvers.

## Author Contributions

UB, DI, GL, and FM were responsible for chemical analyses and tests with plants. NM, EM, and MM designed the experiments, collected bibliography, and performed the statistical analyses. All authors contributed to data analysis and writing of the paper.

## Conflict of Interest Statement

The authors declare that the research was conducted in the absence of any commercial or financial relationships that could be construed as a potential conflict of interest.

## Footnotes

1 www.unep.org

2 https://www.ibm.com/analytics/data-science/predictive-analytics/spss-statistical-software
