# Supplementary material for: Structural and Functional Features of Chars From Different Biomasses as Potential Plant Amendments
Source: Front Plant Sci. 2018 Aug 17;9:1119. doi: 10.3389/fpls.2018.01119 (PMC6108160; doi:10.3389/fpls.2018.01119)
Supplement: Supplementary file 1 [file Data_Sheet_1.PDF]

Supplementary Material

Structural and Functional Features of Chars from Different Biomasses as Potential Plant Amendments

Marta Marmiroli[1], Elena Maestri[1,2*], Urbana Bonas[1], Davide Imperiale[1,3], Giacomo Lencioni[1], Francesca Mussi[2], Nelson Marmiroli[1,2,3]

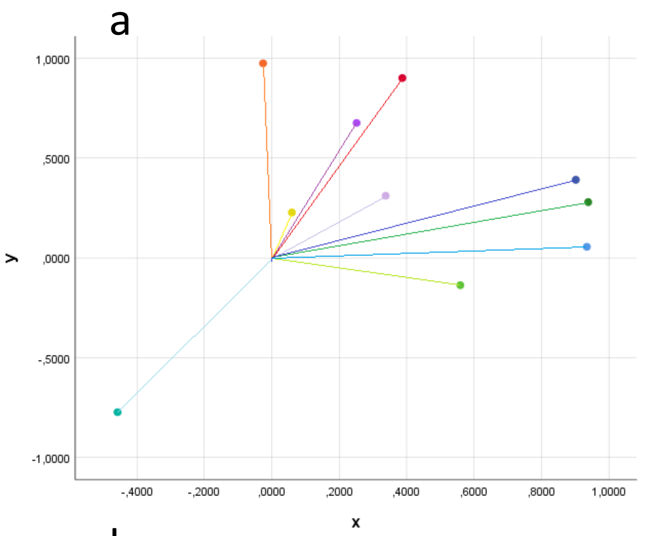

c

Vector	PC1 (x)	PC2 (y)	PC3 (z)
Cd	0.935	0.056	0.340
CE	0.387	0.901	0.129
Cu	0.939	0.279	0.183
Fe	-0.026	0.975	0.020
Ni	0.059	0.227	0.934
OrgSub	-0.458	-0.774	-0.434
Pb	0.338	0.311	0.754
pH	0.251	0.676	0.516
ResHum	0.559	-0.137	0.079
Zn	0.902	0.391	0.173

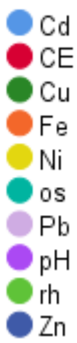

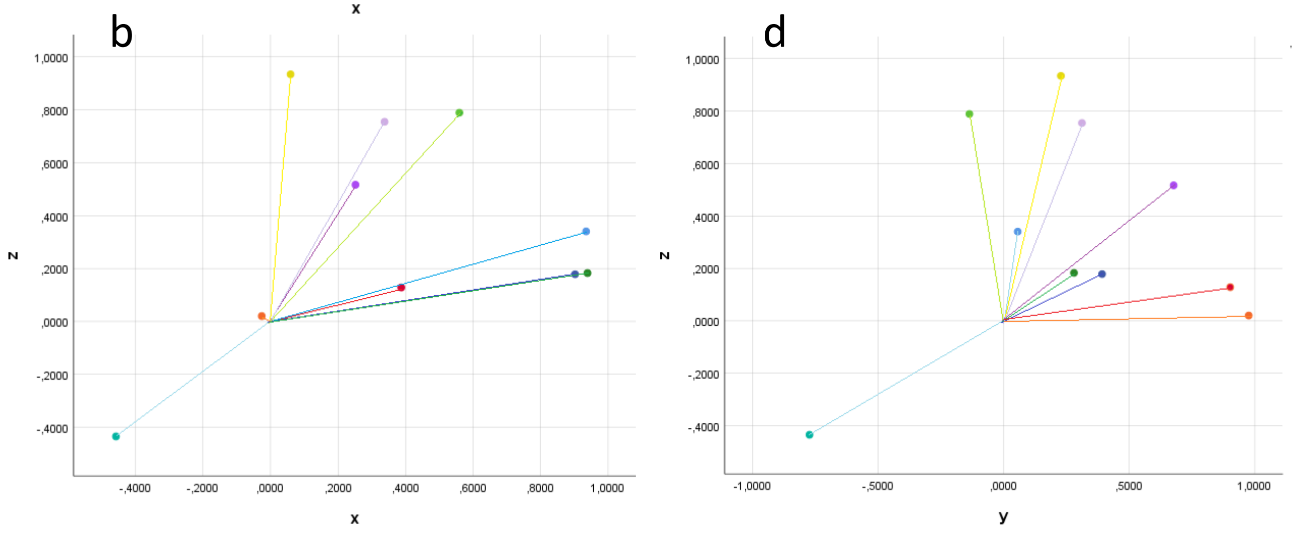

Figure 1. Principal Component Analysis of physico-chemical parameters of chars. All data gathered from all biological replicates of 5 chars, A1, A2, A3, A4 and E1, were analysed using Principal Component Analysis. The legend indicates the colour codes of the different parameters: CE, Electrical Conductivity; os, Organic matter; rh, Moisture Content. (a) First and second PC; (b) first and third PC; (c) vectors loading of all parameters; (d) second and third PC.

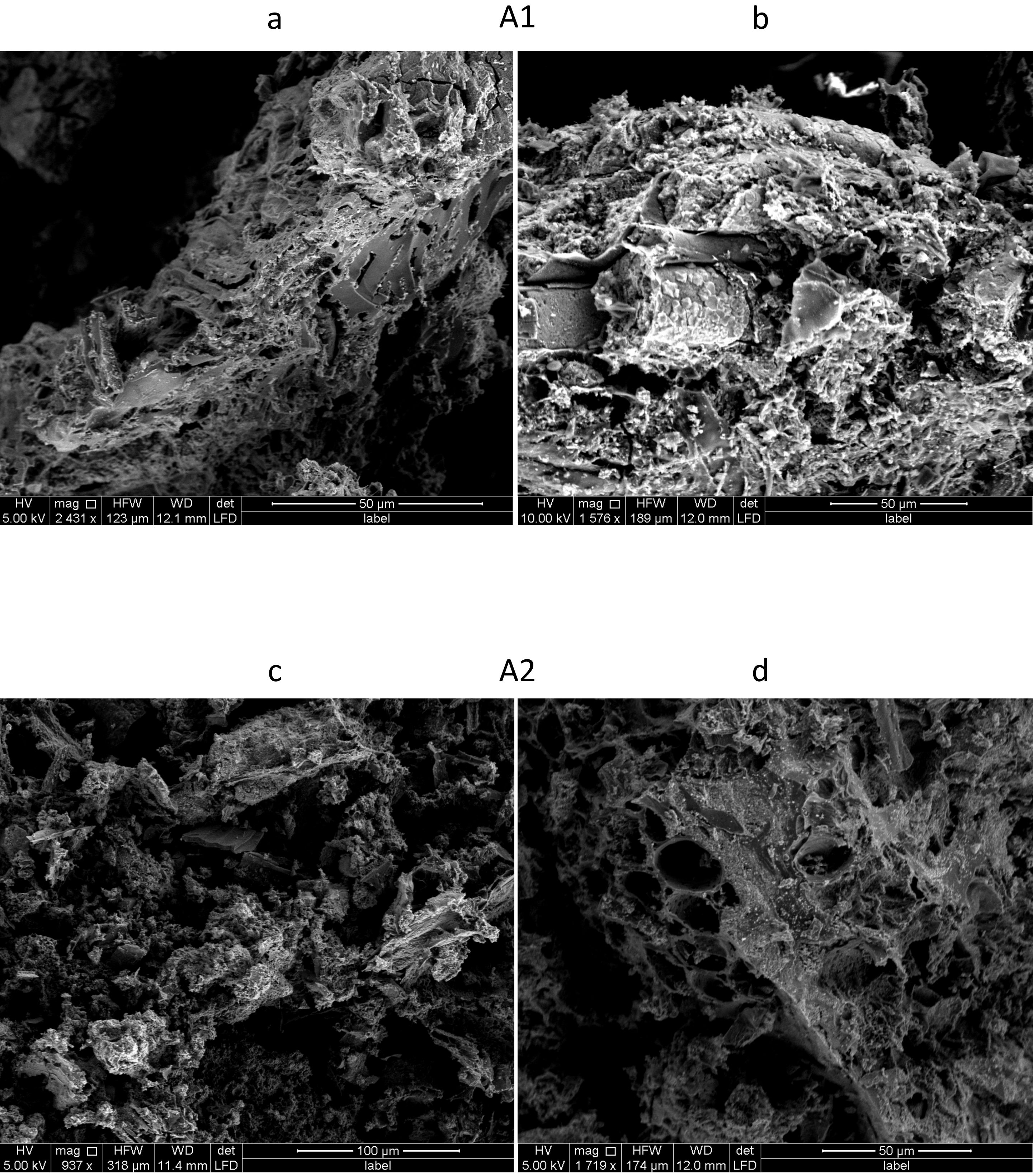

Figure 2. ESEM images of biochars: (a) and (b) A1: (c) and (d) A2 at different magnifications (see figure bottom and white bar bottom right). All images were acquired with electron beam at 5KV and a working distance between 11.9 and 12 mm.

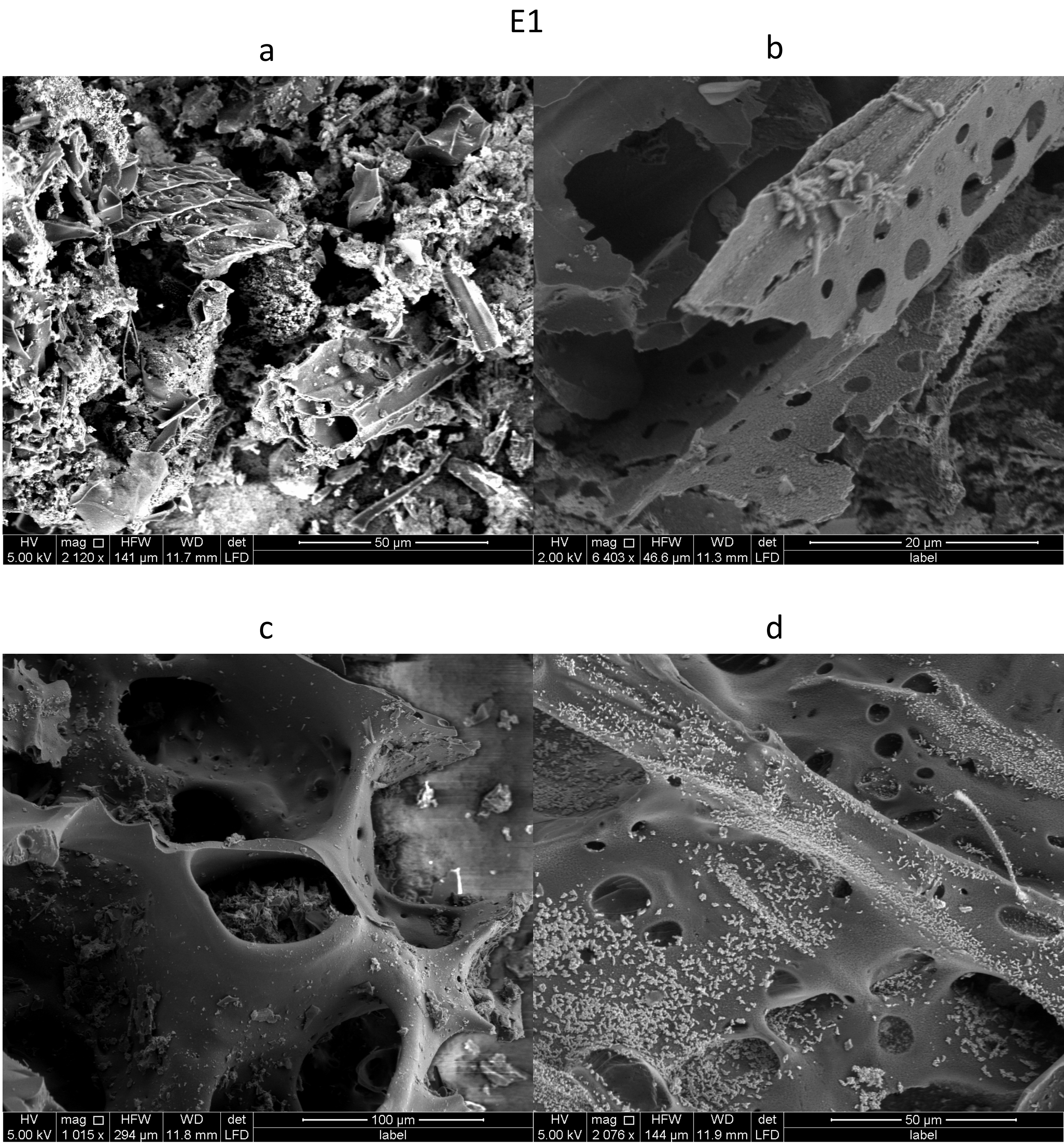

Figure 3. ESEM images of biochar E1 at different magnifications (see figure bottom and white bar bottom right). All images were acquired with electron beam at 5KV and a working distance between 11.9 and 12 mm.

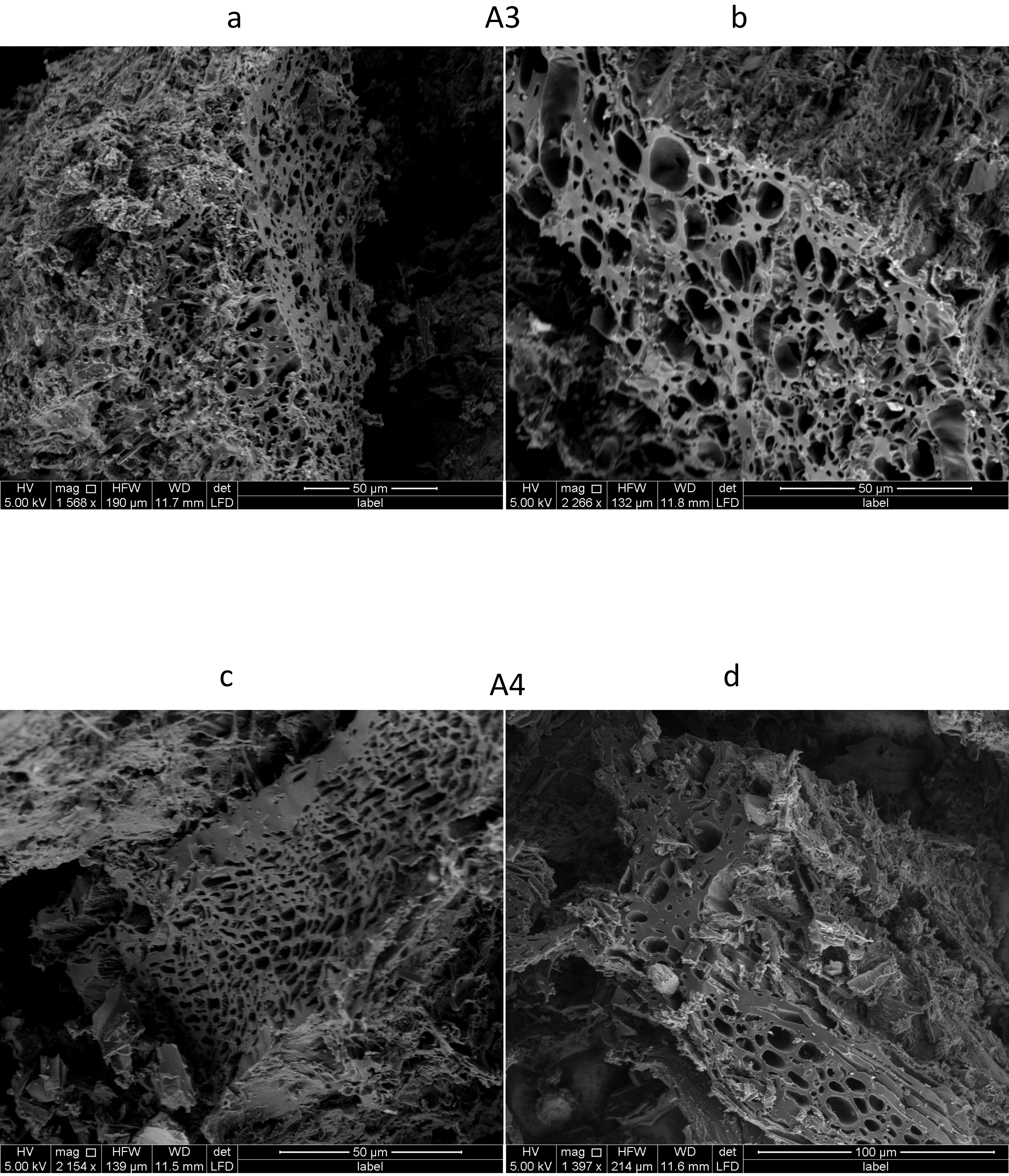

Figure 4. ESEM images of biochars: (a) and (b) A3: (c) and (d) A4 at different magnifications (see figure bottom and white bar bottom right). All images were acquired with electron beam at 5KV and a working distance between 11.9 and 12 mm.

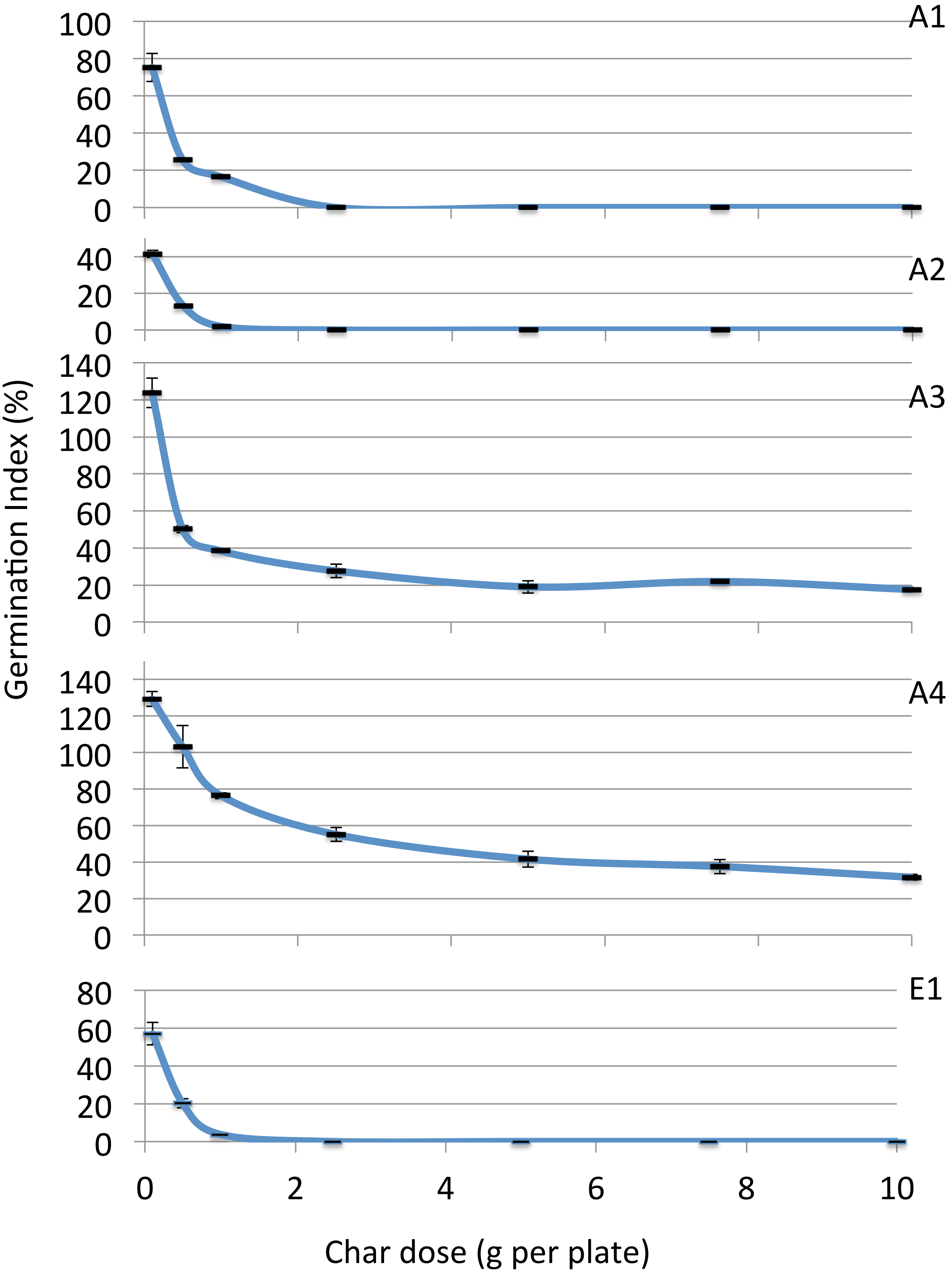

Figure 5. Germination index of Lepidium sativum seedlings germinated for 72 hours in the presence of chars from different feedstocks and production processes at increasing concentrations (0.1 to 10 g per plate). Means of 30 seeds are reported with standard errors. Growth of the control samples in distilled water is considered as 100%.
